# Progenitor cells from limbal, conjunctival and oral mucosal biopsies cultivated on silicone hydrogel contact lenses share CD90, CK15, nestin, CXCR4 and SDF-1 niche markers but preserve tissue-specific characteristics and differentiation directions

**DOI:** 10.3389/fcell.2026.1871330

**Published:** 2026-07-17

**Authors:** Barbara Zsebik, István Rebenku, Gréta Kemenes, Ahmad Nahas, Melinda Vágó, Cameron B. Lloyd, Gergely Losonczy, György Vereb, Lili Takács

**Affiliations:** 1 Department of Biophysics and Cell Biology, Faculty of Medicine, University of Debrecen, Debrecen, Hungary; 2 Hun-Ren-De Cell Biology and Signaling Research Group, Faculty of Medicine, University of Debrecen, Debrecen, Hungary; 3 Faculty of Pharmacy, University of Debrecen, Debrecen, Hungary; 4 Department of Ophthalmology, Faculty of Medicine, University of Debrecen, Debrecen, Hungary; 5 Department of Ophthalmology, Donders Institute for Brain, Cognition and Behaviour, Radboud University Medical Center, Nijmegen, Netherlands

**Keywords:** conjunctival stem cells, explant culture, limbal epithelial stem cell deficiency, limbal epithelial stem cells, niche markers, oral mucosal stem cells, silicon hydrogel contact lenses, whole slide confocal immunofluorescence imaging

## Abstract

**Background:**

Limbal, conjunctival or oral mucosal (OM) epithelial explant cultures are used to treat limbal epithelial stem cell deficiency (LSCD), however, their characteristics remain elusive.

**Methods:**

Explant cultures established on silicon hydrogel contact lenses and source tissue samples were examined by immunofluorescence microscopy for limbal epithelial progenitor, corneal differentiation, and putative limbal niche markers. Quantitative analysis was done using confocal images of whole slides.

**Results:**

In all source tissue samples, many p63+ and some vimentin + basal epithelial as well as CD90+ stromal cells were seen. All epithelia expressed CXCR4. While SDF-1 was mostly in the epithelium and only weakly in the stroma of the limbus and conjunctiva, it was restricted to the stroma in OM. In all explant cultures, vimentin+/p63α+ small cells were abundant. For limbal explants, their ratio was highest at the source (68%) and decreased significantly with distance indicating highest regenerative and differentiation potential. OM showed the lowest ratio (38%) and no change by distance, while conjunctival explants were in the interim range. The density of both corneal-type CK3+ and less differentiated CK19+ epithelial cells increased with distance from the source for limbal and conjunctival samples (R^2^:0.3-0.44, p ≤ 0.05) but did not change for OM (R^2^ < 0.02, p > 0.47). In addition to the dominance of source-specific cytokeratin patterns, all CKs of mature stratified epithelium could be detected, while CK14 and CK15 evidenced the occurrence of less mature epithelial cells. Spatial maturation gradients were only characteristic of limbal sources. Some CD90+ putative niche cells, surrounded by CK15+ and nestin + cells formed distinct clusters in all samples. SDF-1 and CXCR4 were present in all cultures, and their distribution patterns showed similarities with the tissue of origin.

**Conclusion:**

Despite similarities, including putative niche-associated cell clusters, explant cultures on silicon hydrogel surfaces from all three epithelia maintain characteristics of their original source. Their SDF-1 and CXCR4 content indicates the ability to establish contact with the host limbal niche that may be a prerequisite for long-term survival and site-specific differentiation after transplantation. Limbal explants show the highest proliferative and differentiation potential, while the generation of corneal epithelial-like cells is increasingly less efficient from conjunctival and OM sources.

## 1 Introduction

Stem cells of the corneal epithelium reside in the margins of the cornea, the so called limbus (Davanger and Evensen, 1971; Di Girolamo et al., 2015). Limbal epithelial stem cells (LESCs) are in close physical connection with limbal fibroblasts, melanocytes and are surrounded by a microenvironment containing specific growth factors, cytokines and extracellular matrix molecules that regulate the quiescence and proliferation of stem cells (Dziasko et al., 2014; Tseng et al., 2016; Robertson et al., 2021). Altogether these structures make up the limbal epithelial stem cell niche, whose integrity is essential for maintaining epithelial stem cells (Tavakkoli et al., 2023). When LESCs are damaged, a non-transparent and vascularized epithelium develops on the corneal surface, leading to a painful and visually disabling disease, the limbal stem cell deficiency (LSCD). To cure this state, limbal stem cells from the contralateral eye in unilateral cases, or, in bilateral cases, epithelial cells from allogeneic donor eyes or other autologous sources can be transplanted onto the ocular surface (Zhao and Ma, 2015). Oral mucosal (OM) epithelium and conjunctival epithelium are the two alternative autologous sources that have been used so far in clinical practice for LSCD treatment (Utheim, 2013; Inamochi et al., 2019; Sakimoto et al., 2020). In many cases, the relatively simple technique of explant culturing is used for cell amplification before transplantation (Tsai et al., 2000; Ricardo et al., 2013; Kolli et al., 2014; Ramachandran et al., 2014). In explant culturing, a small biopsy sample containing both the epithelium and some subepithelial connective tissue is placed on a carrier surface and cells are allowed to grow in a cell culturing medium (Shortt et al., 2007). Cell outgrowth from such samples involve both epithelial cells and fibroblast-like cell types possibly including niche cells. Besides simplicity, the main advantage of this culturing technique is the independence from mouse fibroblast (3T3) feeder cells that are obligatory components of classic epithelial culturing techniques (Rheinwald and Green, 1975; Pellegrini et al., 1997). Absence of feeder cells and the use of the patients’ own serum instead of fetal calf serum allows animal free culture decreasing the risk of immune reactions and animal virus transmission (Shortt et al., 2007; Kolli et al., 2014; Brejchova et al., 2018).

Many putative limbal epithelial stem/progenitor cell (LESC/LEPC) markers have been identified, including ΔNp63α, CEBPδ, cytokeratins 14, 15, ABCG2, ABCB5 and BMI1 (Di Iorio et al., 2005; Yoshida et al., 2006; Barbaro et al., 2007; Figueira et al., 2007; Takacs et al., 2011; Ksander et al., 2014). Clonogenic conjunctival SCs shown to be present in the lower forniceal and medial canthal areas expressed similar markers: ABCG2 and p63 (Stewart et al., 2015; Bertolin et al., 2021; Bertolin et al., 2022). In the oral mucosa, highly proliferative, CK14 and p63 positive cells are present besides quiescent stem cells in the most basal epithelial OM layer (Madhira et al., 2008; Jones and Klein, 2013), where putative niche cells also reside, expressing proteins associated with TGF-beta (Andl et al., 2016).

In addition to cadherins, ICAM-1,VCAM-1 and integrin α8 that may participate in limbal niche regulation (Polisetti et al., 2016), the SDF-1-CXCR4 axis has been shown to play a role in establishing connection between LESCs and putative limbal niche cells which express embryonal pluripotency markers such as Nestin, Nanog, OCT4 and SSEA4 (Chen et al., 2011; Xie et al., 2011). Cultured limbal stromal niche cells were positive for Nestin, vimentin, CD90, CD105, SDF, and PDGFRβ (Mariappan et al., 2014; Zhu H. et al., 2022). It appears that fibroblastic cells having close contact with the limbal epithelium are different from cells in the deeper limbal stroma (Li et al., 2014; Funderburgh et al., 2016). A recent study attempted to separate limbal niche cells from other limbal stromal cells and found that limbal niche cells express CD90 whereas stromal melanocytes are positive for CD117 (Polisetti et al., 2022) and these two surface markers were sufficient for establishing pure limbal niche cell cultures. To isolate niche cells, collagenase digestion of the limbal tissue is commonly used, which allows deliverance of the superficial stromal cells. Interestingly, explant cultured samples yield limbal niche cells almost as effectively as dispase-collagenase digested samples (Xiao et al., 2020).

Currently, the cellular composition of explant cultures and its dependence on conjunctival or oral mucosal vs. limbal origin is unclear. It also remains a question whether explant cultures contain niche elements with the capacity to get into contact and replenish the host niche. In the present study, we established explant cultures from human limbal, forniceal conjunctival and oral mucosal samples on Lotrafilcon A contact lenses. Using confocal and whole slide immunofluorescence microscopy, the tissues of origin and the whole-mounts of the cell outgrowths were quantitatively and qualitatively analyzed to characterize cell types in the outgrowths. Our goal was to compare expansion and differentiation characteristics, presence of putative niche- and stem cells as well as to reveal factors that potentially play a role in epithelial - niche interaction during the transplantation procedure. We found that explant cultures on silicon hydrogel surfaces from all three epithelial types contain many highly immature cells exhibiting characteristics of both epithelial and mesenchymal/niche origin, and display differentiation potential characteristic not only of the tissue of origin, but also, in varying degrees, of conjunctivo-limbal and corneal epithelium. In all cultures, clusters of CD90+/CK15+/nestin + cells formed compartments reminiscent of niche structures. On the other hand, SDF-1 and CXCR4 positive cells in the cultures may have the ability to establish contact with the host limbal niche.

## 2 Materials and methods

### 2.1 Cultivation of limbal, conjunctival and oral mucosal biopsies

Tissue harvesting was approved and supervised by the local ethical committee (RKEB 4817-2017 and DE RKEB. IKEB 6631-2023, and County Government Office Permission No.: IX-R-052/00016-28/2012). Fifteen limbal, 12 conjunctival and 5 OM samples were harvested from 8 donors (Supplementary Table S1). From each donor, ½ cornea was frozen for immunohistology, from the rest, the limbal collarette was prepared and 2 mm pieces were affixed to contact lens quadrants (Air Optix Night and Day Aqua, Alcon) with sutures as described (Toth et al., 2017). Thus, 4 tissue culture samples were prepared from each donor cornea. Similarly, a part of each conjunctival and OM sample was kept for immunohistology, and 2 mm biopsy pieces were cut from the rest and 2–4 CL quadrants were prepared from each sample to be processed in tissue culture. Thus, altogether 60 pieces of limbal, 56 pieces of conjunctival and 22 pieces of oral mucosal tissue culture on CL quadrants were initiated. Contact lens quadrants were placed into 4-well multidishes (Thermo Fisher Scientific, United States) and grown in a humidified atmosphere with 5% CO2 at 37 °C in a 1:1 mixture of Dulbecco’s minimal essential medium and Ham’s F-12 (DMEM:F-12) supplemented with 10% fetal calf serum (FCS, Sigma-Aldrich, United States), 10 μg/ml insulin (Sigma-Aldrich, United States), 10 ng/ml EGF (Sigma-Aldrich, United States) and 0.5 μg/ml hydrocortisone and antibiotics. On every second day the half amount of medium was changed. After 2-3 weeks, culturing was stopped and outgrowths were fixed and labelled in further experiments. Outgrowth was detected in 45 limbal, 50 conjunctival and 16 oral mucosal samples. For each antibody combination at least two quadrant CLs were used from two different donors. Where quantitative analysis was done, we have used for any given combination of labels one outgrowth each from three sets of matched limbal, conjunctival and OM sources derived from three different donors processed in independent experiments.

### 2.2 Immunofluorescence staining

Cadaveric corneas, conjunctival and oral mucosal samples were embedded in Cryomatrix (Thermo Fisher Scientific, United States), frozen in liquid isopentane chilled with liquid nitrogen, and then stored at −80 °C. Cryostat sections (10–15 µm) were placed on electrostatically charged slides (Superfrost Ultra Plus, Thermo Scientific, United States). Sections were fixed in ice cold acetone and air dried. Cell outgrowths on quarter contact lenses were washed in PBS, fixed with increasing concentrations (10%, 20%, 40%, 70% and 100%) of acetone on Superfrost Ultra plus slides as whole mounts, and stored frozen at −30 °C. Before immunofluorescence staining, sections were rehydrated in PBS containing 2% normal donkey serum and 0.1% nonionic surfactant (Triton X-100, Sigma-Aldrich, United States) for 1 h at room temperature. All samples were incubated at 4 °C overnight with 50 µl primary antibody mixture diluted in PBS containing 2% normal donkey serum and 0.1% nonionic surfactant. The following primary antibody dilutions were used: goat anti-ΔNp63α/TAp63α (hereafter p63α) (Everest Biotech Ltd., United Kingdom Cat# EB10765, RRID: AB_3741577) 1:200; rabbit anti-CXCR4 (Thermo Fisher Scientific Cat# PA3-305, RRID:AB_2091817) 1:100; mouse anti-SDF-1 (Abcam Cat# ab89321, RRID:AB_2049552) 1:100; guinea pig anti-CK3 (Fitzgerald Industries International Cat# 20R-2642, RRID:AB_11191108) 1:100; mouse anti-CK4 (Abcam Cat# ab9004, RRID:AB_306932) 1:250; guinea pig anti-CK12 (LS Bio Cat# LS-C193765, RRID: AB_3741578), 1:100; mouse anti-CK13 (Thermo Fisher Scientific Cat# MA1-35542, RRID:AB_1076277) 1:50; goat anti-CK13 (Everest Biotech Cat# EB09037, RRID:AB_2134700) 1:200; rabbit anti-CK14 (Abcam Cat# ab51054, RRID:AB_869858) 1:50; rabbit anti-CK15 (Novus Cat# NBP2-67525, RRID:AB_2889827) 1:200; Alexa Fluor 647 conjugated rabbit anti-CK19 (Abcam Cat# ab205446, RRID:AB_3674091) 1:20; Cy3 conjugated mouse anti-vimentin (Sigma-Aldrich Cat# C9080, RRID:AB_259142) 1:200; Alexa Fluor 647 conjugated mouse anti-CD90 (BioLegend Cat# 328116, RRID:AB_893430) 1:200, and goat anti-nestin (Thermo Fisher Scientific Cat# PA5-47378, RRID:AB_2609217) 1:200. Samples were washed three times in 0.05% Triton X-100/PBS and then incubated for 1.5 h at room temperature with 50  μl s antibody cocktail (2% normal donkey serum, 0.1% Triton X-100 in PBS) containig 1 μg/ml of one or more of the following donkey antibodies as appropriate: Alexa Fluor 488 anti-rabbit (Thermo Fisher Scientific Cat# A-21206, RRID:AB_2535792); Alexa Fluor 555 anti-rabbit (Thermo Fisher Scientific Cat# A-31572, RRID:AB_162543); Alexa Fluor 647 anti-rabbit (Thermo Fisher Scientific Cat# A-31573, RRID:AB_2536183); Alexa Fluor 488 anti-mouse (Thermo Fisher Scientific Cat# A-21202, RRID:AB_141607); Alexa Fluor 555 anti-mouse (Thermo Fisher Scientific Cat# A-31570, RRID:AB_2536180); Alexa Fluor 647 anti-mouse (Thermo Fisher Scientific Cat# A-31571, RRID:AB_162542); Alexa Fluor 488 anti-goat (Thermo Fisher Scientific Cat# A-11055, RRID:AB_2534102); Alexa Fluor 647 anti-guinea pig (Jackson ImmunoResearch Labs Cat# 706-605–148, RRID:AB_2340476); Alexa Fluor 488 anti-guinea pig (Jackson ImmunoResearch Labs Cat# 706-545–148, RRID:AB_2340472). After checking with all relevant FMO (fluorescence minus one) controls that there is no inter-species cross-binding between primary and secondary antibodies, controls labeled with all relevant secondary antibodies were prepared for every staining experiment. Sections processed identically, but omitting the labels were examined for tissue autofluorescence. All samples were washed three times in 0.05% Triton X-100 in PBS, fixed in 1% PFA/PBS for 10 min, washed again three times, and the cell nuclei were counterstained with 70 µl of 1 μg/ml DAPI for 5 min in the second washing buffer. Sections were mounted in Mowiol 4-88 (Polysciences, Warrington, PA).

### 2.3 Microscopy

Confocal microscopy imaging of tissue sections and contact lens whole mounts was done with a Pannoramic Confocal (3DHistech, Budapest, Hungary) digital pathology scanner with multiplexed confocal fluorescent detection as described earlier (Rebenku et al., 2023). The built-in autofocus algorithm was used with a 20× Plan-Apochromat (NA = 0.8, Carl Zeiss, Jena, Germany). Thirteen confocal slices were imaged with an 800 nm step size. Average intensity projection of the stacks is shown in the overview images. 3D stacks were analyzed in detail using the SlideViewer (3DHistech, Hungary) application.

High resolution confocal images were obtained with a Zeiss LSM 880 confocal laser scanning microscope (Carl Zeiss, Jena, Germany). Detection was through a water immersion 40x objective (NA = 1.2) with a 32 element GaAsP array integrating the relevant spectral bands beyond a dispersion grating. To avoid crosstalk, each fluorophore was excited and detected separately. Pinhole setting was 1.7–2.6 Airy Units, and Nyquist sampling criteria were used throughout.

### 2.4 Cell counting in whole mounts

For image analysis ImageJ 1.53 h (bundled in Fiji) (Schindelin et al., 2012) and QuPath 0.2.3 (Bankhead et al., 2017) were used. The field of view was divided into 1 mm square tiles (ROIs) for quantification. Cells were identified and counted using QuPath’s built-in supervised pixel classifier machine learning algorithm. The DAPI channel was used for training (Classify ‣ Pixel classification ‣ Train pixel classifier) by pointing at positive and negative pixels in the ROI while “live prediction” was “on” to enable continuous training until all visually identifiable nuclei were correctly classified. At this point identified nuclei were created as objects. Specific immunofluorescent labelling of each cell that belonged to an identified nucleus was visually assessed to exclude artefacts originating from partially overlapping cells in different layers. Cells positive (or double positive) for the label(s) of interest were counted in ROIs randomly chosen so as to cover various distances from the explant source. The distance of the center of weight of the source and of the ROI was measured, and ROI position was classified as near (0–2 mm), middle (2–4 mm) or far (4–7.9 mm).

### 2.5 Statistical analysis

GraphPad Prism 5 software (GraphPad software, Inc., La Jolla, CA) was used for statistical analysis. As more than two groups have been compared, one-way or two-way ANOVA was used after confirming normality with a Shapiro-Wilk test. For unequal variances, Brown Forsythe and Welch correction was applied. Pairwise comparison was done with Bonferroni’s *post hoc* test. The critical level of significance was set to α = 0.05. Cell count as a function of distance from the source was analyzed with linear regression. Data are presented as mean ± SD as error bars for n ≥ 3 donors.

### 2.6 Institutional review board statement

The study was conducted in accordance with the Declaration of Helsinki, and tissue harvesting was approved and supervised by the Institutional ethical committee of the University of Debrecen (County Government Office Permission No.: IX-R-052/00016-28/2012, RKEB 4817-2017 and DE RKEB.IKEB 6631-2023).

## 3 Results

**FIGURE 1:**
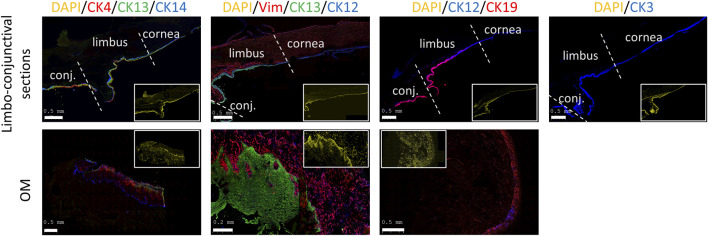
Localization of cytokeratins (CK) in limbal, conjunctival and oral mucosal (OM) epithelia. Radial sections spanning the conjunctiva, limbus and the cornea (top row, regions separated by indicator lines) as well as sagittal sections of the OM (bottom row) were immunostained for CK3, CK4, CK12, CK13, CK14, CK19 and vimentin as indicated by the labels and counterstained with DAPI. Average intensity projections of confocal stacks from whole slides are presented with DAPI images of nuclei as separate inserts for better visibility of the immunofluorescent signal. Scale bars: 0.5 mm CK4 is located in the superficial layers of the conjunctival, limbal corneal and OM epithelia. CK14 is present in the basal layer of all epithelial types. CK13 is present in all layers of the OM epithelium and in the middle layers of conjunctival, limbal and corneal epithelia. CK12 is only seen in the corneal and limbal epithelia. CK19 is present in limbal, conjunctival and OM epithelia, but absent from the cornea. CK3 is seen in the limbus, cornea and also in the conjunctiva.

**FIGURE 2:**
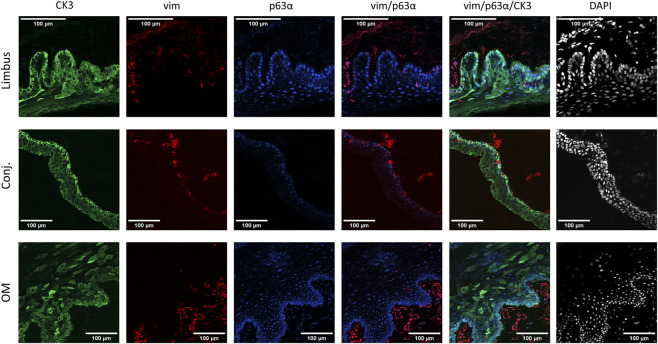
CK3, vimentin and p63α staining in limbal, conjunctival and OM epithelia. High resolution confocal images were acquired after immunofluorescent labeling of cryosections. Individual optical channels and their combinations are presented, as well as DAPI nuclear staining images separately for better visibility. Scale bars: 100 μm. CK3 is present in all epithelial types. The basal layers of all epithelia contain p63α brightly positive cells. In addition to the stromal cells, some basal epithelial cells are vimentin positive in all epithelia.

In tissue sections, corneal epithelium was positive for CK3 and CK12 in the upper layers and CK14 in basal layers. Limbal epithelium contained CK3 and CK12 as well as CK13, CK4, CK14, and mostly CK19. Conjunctival epithelium was positive for CK19, CK13 and CK14, and, also weakly for CK3. OM was positive for CK13, CK4, CK14, CK19, and some cells also for CK3 (Figures 1, 2). The basal epithelial layer of all tissues contained vimentin positive cells (Figure 2) vimentin positive cells were located in the vicinity of brightly p63 positive basal epithelial cells (Figure 2). In the conjunctiva and limbus, vimentin positive cells were present in the basal epithelial layer, whereas in the OM, mostly on the stromal side, adjacent to them (Figure 2).

**FIGURE 3:**
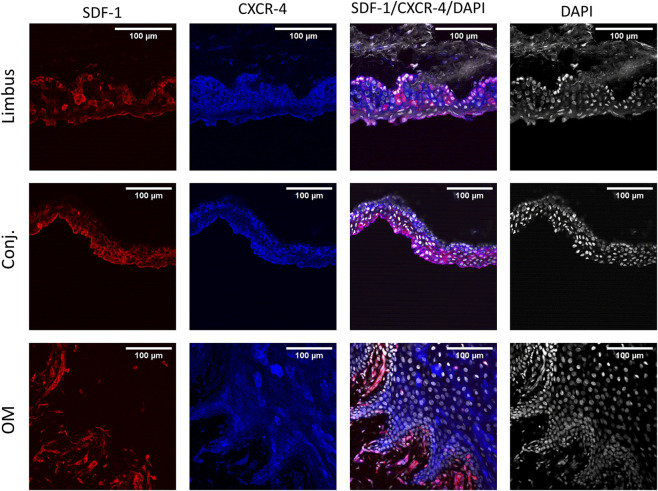
SDF-1 and CXCR4 staining in limbal, conjunctival and OM epithelia. High resolution confocal images were acquired after immunofluorescent labeling and DAPI nuclear counterstain of cryosections. Individual optical channels and their combinations are presented. Scale bars: 100 µm. SDF-1 and CXCR4 are present in limbal and conjunctival epithelia, where a lot of epithelial cells are double-positive. In the OM, SDF-1 is mostly localized to stromal cells and CXCR4 to epithelial cells double positive cells in the epithelium are scarce.

SDF-1 and CXCR4, a receptor-cytokine pair that has been suggested to play a role in establishing the connection between limbal niche cells and epithelial cells, was also found in all three tissues. In the conjunctiva and limbus, both SDF-1 and CXCR4 staining was strongly positive in epithelial cells and SDF-1 was weekly positive in stromal cells. However, in the OM, SDF-1 was only present in stromal cells, whereas CXCR4 was found in the epithelial layers. (Figure 3).

**FIGURE 4:**
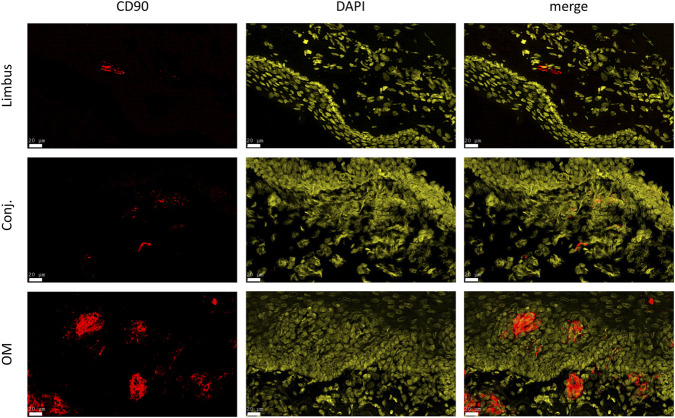
CD90 expression patterns in limbal, conjunctival and OM epithelia. High resolution confocal images were acquired after immunofluorescent labeling and DAPI nuclear counterstain of cryosections. Individual optical channels and their overlay are presented. Scale bars: 20 µm. In the limbus and conjunctiva, rare CD90+ cells are visible in the subepithelial stroma, in the OM, clusters of CD90+ cells are located in the stromal projections within the thick epithelium.

CD90+ putative niche-associated stromal cells were present in the subepithelial stroma of limbal, conjunctival and OM samples. While in the limbus and conjunctiva these cells were rare and scattered, they formed abundant clusters in the stromal projections of OM (Figure 4).

**FIGURE 5:**
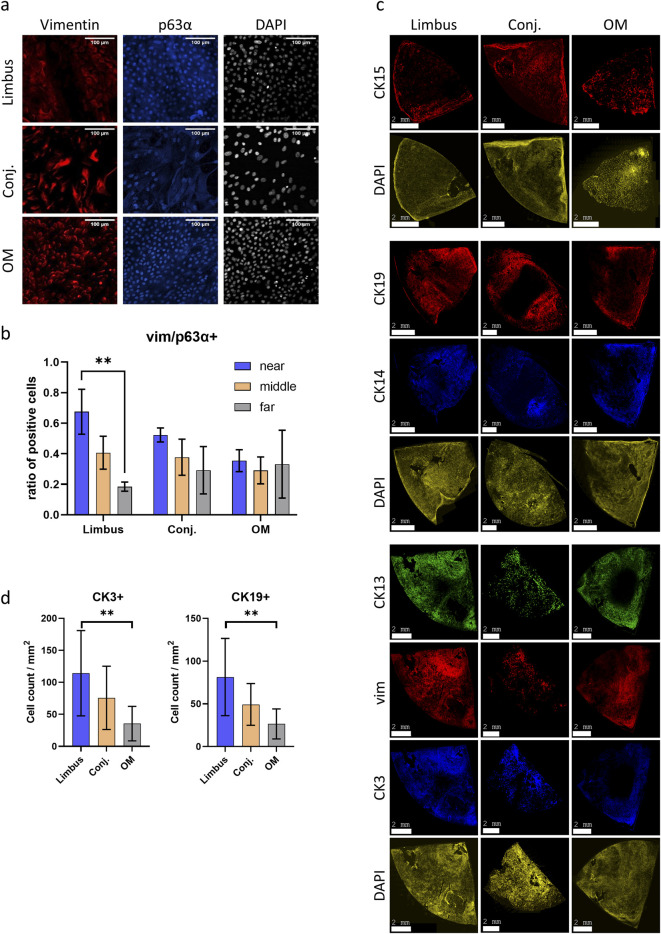
Cytokeratin, vimentin and p63α expression in outgrowths from limbal, conjunctival and OM biopsies. Sets of matching limbal, conjunctival and OM biopsies were obtained from three donors and used throughout these experiments. **(a)** Vimentin and p63α staining in high resolution confocal images of cultured cell sheets on contact lens surfaces at middle distance from the explant source. Scale bars: 100 µm. All sheets contain small, vimentin positive cells, with p63α nuclear staining. From the conjunctival source, many vimentin + cells are elongated, while in OM and limbal samples small, round cells dominate. **(b)** Quantitative analysis of vimentin and p63α positive cells as a function of distance from the biopsy source. Matching limbal, conjunctival and OM outgrowths for 3 independent donors were labeled. In each image, for each distance category (near, middle and far), the average ratio of double positive cells to total nuclei was determined from three randomly chosen ROIs. For each source tissue and distance category, the mean ± SD of these average ratios from the three donors are displayed (n = 3, **p < 0.01). Limbal explants produce the largest proportion of undifferentiated vim+/p63+ cells close to the source and their presence significantly decreases far from the explants. A similar, but not significant tendency is seen in conjunctival samples, whereas the lowest proportion and no decreasing tendency is observed in OM. **(c)** Cytokeratin patterns in whole mount outgrowths on contact lenses. Average intensity projections of confocal stacks from whole slides are presented, displaying in separate channels the cytokeratins labeled each sample and the corresponding DAPI nuclear counterstain in the bottom row of each block. Scale bars: 2 mm. Larger, cytokeratins positive cells in the cultures express a repertoire of various CKs. Cornea specific CK3 is also present in conjunctival and OM outgrowths, while CK14 and CK15 characteristic of less differentiated, and CK13 and CK19 of more differentiated stages of nonkeratinizing stratified epithelia are ubiquitous in all explant cultures. **(d)** Quantitative analysis of the density of CK19 and CK3 positive cells in outgrowths. Matching limbal, conjunctival and OM outgrowths for 3 independent donors were labeled. In each image, CK3 and CK19 positive cells were counted in ≥3 randomly chosen ROIs that were far (≥4 mm) from the source. For each source tissue the mean ± SD of cell density is displayed (n = 3 independent donors, N ≥ 9 ROIs, **p < 0.01). Conjunctivo-limbal specific CK19+ and corneal specific CK3+ cells are significantly more abundant in limbal than in OM cultures.

**FIGURE 6:**
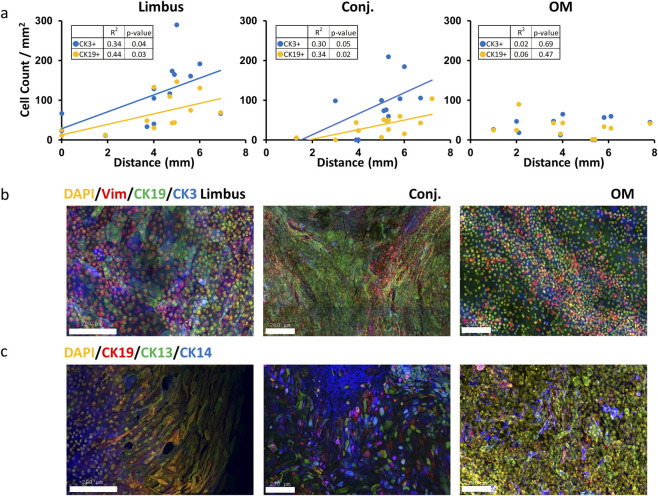
Spatial gradients and patterns of epithelial differentiation of outgrowths on contact lenses. Sets of matching limbal, conjunctival and OM biopsies were obtained from three donors and used throughout these experiments. Cytokeratins 3, 13, 14 and 19, vimentin and DAPI were labeled and imaged in whole mount outgrowths on contact lenses. **(a)** In each whole mount image, CK3 and CK19 positive cells were counted in ≥11 randomly chosen ROIs. Cell count/mm^2^ was plotted against the distance between the center of weight of the source and of the ROI and linear regression was calculated (n = 3 independent donors, N = 14, 13 and 9 ROIs for limbal, conjunctival and OM explants). In limbal and conjunctival samples, CK3+ and CK19+ cell numbers increase with distance from the explant source. Such correlation is not seen in OM cultures. **(b,c)** Average intensity projections of confocal stacks from whole mounts. Scale bars: 200 μm. **(b)** Among larger, differentiated cells, CK3+ cells are dominant in limbal samples, CK19+ cells in conjunctival samples. **(c)** CK13+ cells are more prevalent in conjunctival and OM samples. Starting from CK14 basal-type cells, directional migration of cells differentiating to CK19 and then to CK13 positivity occurs in limbal samples. Such a pattern was less obvious in conjunctival cell sheets and not visible at all in OM samples.

Cultured cell sheets on contact lens surfaces of all three tissue types contained many small, vimentin positive cells, with p63α nuclear staining. From the conjunctival source, many vimentin + cells were elongated, fibroblast-like, while OM and limbal samples were dominated by small, round cells (Figure 5a). Quantitative analysis of cells from three different donors showed that limbal explants produced the largest proportion of undifferentiated vim+/p63+ cells close to the source (mean ± SD: 68% ± 13%) and their presence significantly decreased far from the explants indicating both the highest proliferative capacity and that cells farther from the source underwent differentiation. A similar, but not significant tendency was seen in conjunctival samples, with a 52% ± 0.04% proportion of vim+/p63+ cells close to the source, whereas the lowest proportion (35% ± 0.06%) and no decreasing tendency were observed in OM (Figure 5b). Besides small vimentin + cells, larger CK + cells were also present in the cultures. These larger cells expressed a repertoire of various cytokeratins, not only CKs specific for the given tissue (Figure 5c). Cornea specific CK3 was also present in conjunctival and OM outgrowths, while CK14 and CK15 characteristic of less differentiated, and CK13 and CK19 of more differentiated stages of nonkeratinizing stratified epithelia were ubiquitous in all explant cultures (Figure 5c). Quantitative analysis showed that conjunctivo-limbal specific CK19+ and corneal specific CK3+ cells were significantly more abundant in limbal than in OM cultures (Figure 5d). Moreover, in limbal and conjunctival samples, CK3+ and CK19+ cell numbers increased as cells migrated away from the explant source, whereas such correlation was not seen in OM cultures (Figure 6a). At the same time, among differentiated cells, CK3+ cells were dominant in limbal samples, CK19+ cells in conjunctival samples (Figure 6b) whereas CK13+ cells were more prevalent in conjunctival and OM samples (Figure 6c). Starting from CK14 basal-type cells, directional migration of cells differentiating to CK19 and then to CK3 positivity occurred in limbal samples, presenting a degree of compartmentalization reminiscent of cellular layering in native limbal tissues. Such a pattern was less obvious in conjunctival cell sheets and not visible at all in OM samples (Figure 6c).

**FIGURE 7:**
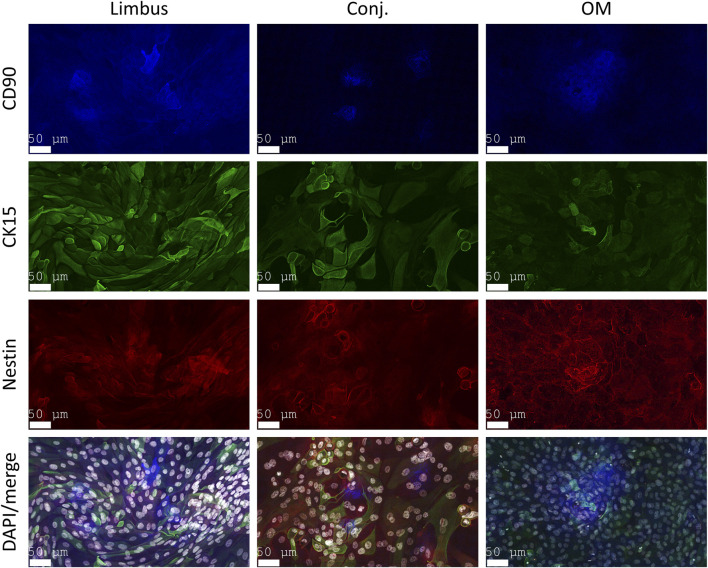
CD90, CK15 and nestin expression patterns in limbal, conjunctival and OM derived outgrowths. Whole mounts were immunostained and counterstained with DAPI. Average intensity projections of confocal stacks from whole slides are presented for each marker separately, and overlaid, together with the nuclear signal. Scale bars: 50 µm. Some of the cultured CD90+ cells are organized into niche-like domains with nestin+ and CK15+ cells surrounding them. Notably, CD90+ cells occur as groups of single cells in limbal and conjunctival cultures, whereas they form clusters in OM samples.

In every sample from all three tissue types, some of the small vim + cells were CD90+ and were organized into domains with nestin+ and CK15+ cells surrounding them, reminiscent of niche structure. Notably, CD90+ cells occurred as groups of single cells in limbal and conjunctival cultures, whereas they formed clusters in OM samples (Figure 7), this pattern being similar to that occurring in the original tissue samples (Figure 4). These CD90+ groups were present in the proximity or at most at mid-distance from the explant sources.

**FIGURE 8:**
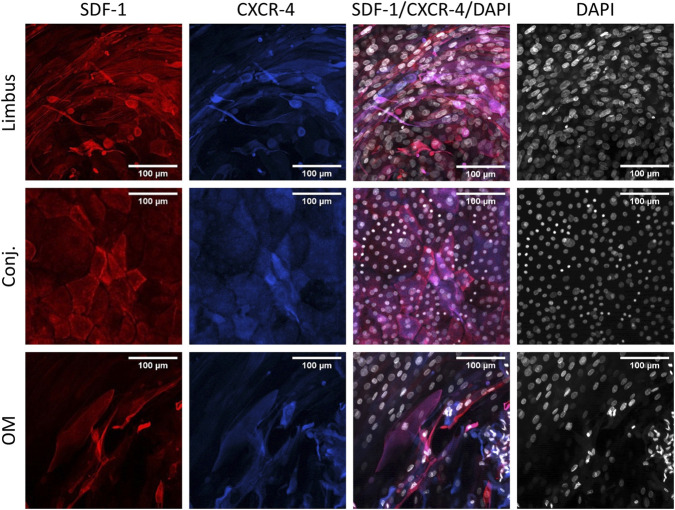
SDF-1 and CXCR4 expression patterns in limbal, conjunctival and OM derived outgrowths. High resolution confocal images from whole mounts were acquired after immunofluorescent labeling and DAPI nuclear counterstain. Individual optical channels are presented for each marker separately, and overlaid, together with the nuclear signal. Scale bars: 100 µm. Cells positive for SDF-1 and CXCR4 are present in all three cultures. In limbal and conjunctival samples, small round cells are mostly SDF-1 positive, but larger CXCR4/SDF-1 double positive cells are also present. In OM, however, double positive cells are rare, similarly to tissue samples.

Cells positive for SDF-1 and CXCR4 were also present in all three cultured cell layers (Figure 8). In limbal and conjunctival samples, small round cells were mostly SDF-1+ whereas larger CXCR4/SDF-1 double positive cells were also present. In OM, however, double positive cells were rare, and similarly to tissue samples, CXCR4 and SDF-1 staining mostly occurred in separate cells.

## 4 Discussion

Various carriers have been used for LESC culturing (reviewed recently by (Di Girolamo, 2024), however only the human amniotic membrane (HAM) (Nakamura et al., 2011; Zakaria et al., 2014) fibrin (Pellegrini et al., 1997; Rama et al., 2010) and Lotrafilcon A contact lenses (CLs) (Di Girolamo et al., 2009; Bobba et al., 2015), and PLGA membrane (Ramachandran et al., 2021) have been used in clinical trials. All of these carriers were proven to enable LSCT with success rates between 46.7% to 90.9% (reviewed by (Genna et al., 2025)). However, the success of LSCT may decrease on the long term (Behaegel et al., 2019) and one proposed way to increase success is the regeneration of the limbal niche (Bonnet et al., 2024). In animal models, transplantation of *ex vivo* cultivated, collagenase digested limbal tissue was used for transplantation of limbal epithelial and niche cells (Higa et al., 2020), where a single organoid was sufficient to restore corneal epithelium, moreover, limbal niche cells transplanted together with epithelial progenitor cells ensured better epithelial maintenance and stromal transparency (Zhu L. et al., 2022). In a clinical trial, cultured collagenase digested oral mucosal cell sheets containing epithelial and niche cells showed long-term survival when transplanted onto LSCT ocular surface (Ma et al., 2021).

Previously, we have successfully established explant cultures of limbal, conjunctival, and OM samples on Lotrafilcon A CLs (Toth et al., 2017; Zsebik et al., 2017). In previous clinical trials, it was shown that transplantation results were better when the tissue explants affixed to CLs remained on the recipient corneal surface (Di Girolamo et al., 2009; Bobba et al., 2015). Since niche cells were shown in explant cultures (Xiao et al., 2020), moreover, increased stem cell outgrowth was detected in long-term passaging of explant cultures (Gurdal et al., 2025), it can be assumed that niche cells released from the explants may contribute to better survival of transplanted epithelial sheets on Lotrafilcon A CLs, especially when explants come into contact with the corneal surface. When alternative epithelia are used for LSCT, the presence of CK12 containing corneal-type epithelial cells is crucial to restore corneal clarity, and the presence of such cells was shown after successful corneal surface restauration by conjunctival and OM epithelial transplants (Yamagami et al., 2018; Prabhasawat et al., 2021; Attico et al., 2023). In this study, we examined differentiation characteristics and putative niche cell markers in explant cultured limbal, conjunctival and OM samples on CLs to explore whether they bear the potential to differentiate into corneal-type epithelium and if they can contribute to limbal niche restauration.

With the digital confocal pathology scanner used in this study, the whole outgrowth can be visualized, thus we could observe specific patterning of outgrowths. In explant cultures on CL surfaces from conjunctival, limbal and oral mucosal epithelial samples, we observed a massive outgrowth of small vimentin and p63α positive cells. The density of p63+ cells was highest in limbal samples, and rapidly and significantly decreased as the distance increased from the explant source. A lower density and steady but non-significant decrease were observed in conjunctival samples, while OM gave rise to the lowest density that showed no decrease over distance. Similarly, the amount of CK3 and CK19 positive cells increased in limbal and conjunctival samples as the distance from the source increased, whereas no such correlation was observed in OM samples. These collectively indicated that differentiation occurred as cells migrated farther away from limbal and conjunctival sources, and that both regenerative and differentiation ability decreases from limbal through conjunctival to OM explant cultures.

In addition to cornea-specific CK3 and conjunctivo-limbal specific CD19, in all tissue outgrowths a number of cells were positive for cytokeratins CK14 and CK15, characteristic of less differentiated basal layers of stratified epithelia. Our results support previous findings of CK3+ cells in OM and conjunctival cultures (Sharma et al., 2012; Ricardo et al., 2013; Toth et al., 2017) and are in accordance with our observation that rare CK3+ cells are present in OM and conjunctival tissues (Figures 1, 2). However, it is possible that in culture, differentiation cues arriving from stromal cells are less abundant, thus a variable, not only tissue specific epithelial differentiation may occur. In accordance, when no such cues are present after limbal injury, epithelial cells were shown to randomly differentiate into CK12, CK10 or CK13 containing epithelia or even goblet cells (Park et al., 2022).

In our samples, epithelial cells grew together with their vimentin + stromal cells, thus the presence of differentiation cues could be assumed. In accordance with this assumption, tissue specific CKs (CK3 in limbal, CK19 in conjunctival and CK13 in OM samples) were the most abundant besides other types. Moreover, in limbal samples compartmentalization of CK14+ cells was observed, with CK19+ and CK3+ cells migrating unidirectionally away from them, similar to the organization of limbal layers. Some compartmentalization without obvious directional migration was seen in conjunctival outgrowths and there was no compartmentalization in OM. A similar self-organization of explant cultured limbal epithelia on HAM was shown earlier, manifesting in whirl-like epithelial structures indicating a “differentiation memory” of the cultures (Mariappan et al., 2014).

To the authors’ best knowledge, the presence of the CXCR4-SDF-1 cytokine-receptor pair has not been previously examined in explant cultures. We found that patterning of CXCR4 and SDF-1+ cells in the outgrowths was also reminiscent of the tissue of origin. CXCR4-SDF-1 signaling in the limbus was shown to be necessary for niche cell-LESC connection, and niche cell regeneration (Xie et al., 2011; Chen et al., 2021). However, in the conjunctiva and OM, CXCR4-SDF-1 signaling was primarily associated with fibrotic changes, such as pterygium formation and development of oral submucous fibrosis (Bamdad et al., 2017). Nevertheless, expression of CXCR4 on OM and conjunctival cell outgrowths suggests the possibility of interaction between these cells and limbal niche cells. Cumulatively, our findings indicate that the CXCR4–SDF-1 axis may play an important role in the re-establishment of niche–epithelial interactions in the LSCT procedure. However, as our work is primarily descriptive and confirms only the presence of this signaling pathway, further functional studies are required to elucidate its precise role. Such investigations may ultimately reveal novel therapeutic possibilities for LSCT. Herein, an important aspect is the modulation of inflammation by CXCR4 signaling (Ratajczak et al., 2006; Lopez et al., 2018), which highlights the necessity of suppressing inflammation in LSCD patients by HAM transplantation or long-term use of steroid eye drops (Tseng et al., 2016). Additional options that have recently emerged are microvesicle-containing eye drops (Pedersen et al., 2024) and mesenchymal (stem) cell transplantation (Meshko et al., 2024).

We also examined the presence of CD90+ putative niche-associated stromal cells in the cultures. To our knowledge, this study is the first to observe CD90+ stromal cell population in limbal, conjunctival and OM explant outgrowths. Earlier, CD90 was shown in OM mesenchymal cells (Matsumura et al., 2015) and isolated CD90+ OM stromal cells supported OM epithelial growth in cell culture (Kriegebaum et al., 2012). CD90+ cells were shown in porcine subconjunctival stroma, but their role has not been further elucidated (Polisetti et al., 2023). In our experiments, CD90+ cells were spatially associated with nestin + cells and basal epithelial-like CK15+ cells in all tissue samples, in clusters reminiscent of niche-like stromal structure. Although CD90 positivity in limbal stromal cells was sufficient to isolate niche cells in one study (Polisetti et al., 2022), at present, CD90 is not an established niche-specific marker and is broadly expressed by fibroblasts, mesenchymal stromal cells, and activated stromal cells. Therefore, although the observed spatial organization suggests a potential association with epithelial support functions, the present findings are insufficient to definitively establish a niche-supportive role for these cells. Furthermore, given the explant culture setting, stromal cell expansion and culture-induced phenotypic alterations cannot be excluded. On the other hand, the presence of these stromal populations together with undifferentiated epithelial-like cells in cultures may suggest that long-term survival of epithelial stem cells in this system is possible, similarly to the long-term survival of OM cells up to 10 years after COMET observed in transplanted patients (Attico et al., 2023). This also emphasizes the potential of the host’s niche restauration and the recruitment of host LSC by transplanted cell sheets, since disappearance of donor cells and repopulation only by host corneal cells was observed after LSCT (Daya et al., 2005; Chotikavanich et al., 2023).

Finally, our results indicate that Lotrafilcon-A contact lenses provide an excellent surface for explant culturing of limbal, conjunctival and OM stem cells, maintaining large numbers of immature cells, and also some putative niche characteristics. Lotrafilcon-A contact lenses have been used successfully as a carrier for LSCT (Bobba and Di Girolamo, 2016). Besides, they are easy to handle and transparent, thus can be used to study the properties of explant cultured cells. In the present study, this culture technique made possible observations that suggest a role for better preservation of putative niche cells and reinforcement of niche-epithelial interactions via the CXCR4-SDF-1 signaling in increasing the success of limbal stem cell transplantation.

Limitations of our study include, firstly, that the experiments were performed in an *ex vivo* explant culture system, which cannot fully reproduce the complexity of the native limbal microenvironment or post-transplantation conditions. Second, the study is primarily descriptive and based mainly on immunofluorescent characterization. While our *in situ* analysis of contact lenses provides some insight into proliferative ability through the proportion of P63+/Vim + cells, and characterize the cells’ ability to differentiate and migrate via the correlation between the density of differentiated CK3+ and CK19+ cells and the distance from the source, these are only surrogate markers that could prospectively be supported by classical colony-forming and migration assays, and, particularly, transplantation studies further validating functional implications. Notably, the potential role of the CXCR4-SDF1 axis could be further examined by migration assays, receptor-blocking experiments, and co-culture validation studies to substantiate its role in LSCT. Finally, the relatively limited donor number should also be considered when interpreting the statistical analyses and generalizability of the findings.

## Data Availability

The raw data supporting the conclusions of this article will be made available by the authors, without undue reservation.
