# Supplementary material for: Progenitor cells from limbal, conjunctival and oral mucosal biopsies cultivated on silicone hydrogel contact lenses share CD90, CK15, nestin, CXCR4 and SDF-1 niche markers but preserve tissue-specific characteristics and differentiation directions
Source: Front Cell Dev Biol. 2026 Jul 17;14:1871330. doi: 10.3389/fcell.2026.1871330 (PMC13424239; doi:10.3389/fcell.2026.1871330)
Supplement: Supplementary file 1 [file DataSheet1.pdf]

Age	Gender	OD/OS	Sample type	Histology	Cultivation
40	female	OS	limbus	yes	no
81	female	OD	whole cornea	yes	no
	female	OD	whole cornea	yes	no
80	female	OD/OS	conjunctiva, cornea, OM	yes	yes
62	female	OD/OS	corneolimbal transition, limbus, OM	yes	yes
	male	OD/OS	conjunctiva, corneolimbal transition, OM	yes	yes
70	male	OS	limbus, central cornea	yes	yes
60	male	OD/OS	conjunctiva, limbus	yes	yes
91	male	OD/OS	conjunctiva, limbus	yes	yes
67	male	OD/OS	conjunctiva, corneaolimbal transition, central cornea, OM	yes	yes
61	female	OD/OS	conjunctiva, OM, cornea	yes	yes
